# A multipurpose machine learning approach to predict COVID-19 negative prognosis in São Paulo, Brazil

**DOI:** 10.1038/s41598-021-82885-y

**Published:** 2021-02-08

**Authors:** Fernando Timoteo Fernandes, Tiago Almeida de Oliveira, Cristiane Esteves Teixeira, Andre Filipe de Moraes Batista, Gabriel Dalla Costa, Alexandre Dias Porto Chiavegatto Filho

**Affiliations:** 1 School of Public Health, University of São Paulo, São Paulo, SP Brazil; 2 Fundacentro, São Paulo, SP Brazil; 3 Statistics Department, Paraíba State University, Paraíba, PB Brazil; 4 Bioinformatics and Computational Biology Lab, Brazilian National Cancer Institute, Rio de Janeiro, RJ Brazil; 5 BP-A Beneficência Portuguesa de São Paulo, São Paulo, SP Brazil

**Keywords:** Computational biology and bioinformatics, Machine learning, Predictive medicine

## Abstract

The new coronavirus disease (COVID-19) is a challenge for clinical decision-making and the effective allocation of healthcare resources. An accurate prognostic assessment is necessary to improve survival of patients, especially in developing countries. This study proposes to predict the risk of developing critical conditions in COVID-19 patients by training multipurpose algorithms. We followed a total of 1040 patients with a positive RT-PCR diagnosis for COVID-19 from a large hospital from São Paulo, Brazil, from March to June 2020, of which 288 (28%) presented a severe prognosis, i.e. Intensive Care Unit (ICU) admission, use of mechanical ventilation or death. We used routinely-collected laboratory, clinical and demographic data to train five machine learning algorithms (artificial neural networks, extra trees, random forests, catboost, and extreme gradient boosting). We used a random sample of 70% of patients to train the algorithms and 30% were left for performance assessment, simulating new unseen data. In order to assess if the algorithms could capture general severe prognostic patterns, each model was trained by combining two out of three outcomes to predict the other. All algorithms presented very high predictive performance (average AUROC of 0.92, sensitivity of 0.92, and specificity of 0.82). The three most important variables for the multipurpose algorithms were ratio of lymphocyte per C-reactive protein, C-reactive protein and Braden Scale. The results highlight the possibility that machine learning algorithms are able to predict unspecific negative COVID-19 outcomes from routinely-collected data.

## Introduction

The consequences of a long stay and demand for hospital resources due to COVID-19 have been disastrous for health systems in middle and low-income countries (LMICs)^1,2^, requiring immediate clinical decisions, especially when dealing with limited resources^3,4^. An accurate COVID-19 prognosis assessment is crucial for screening and treatment procedures and may increase patient survival^5,6^. In Brazil^7^, many cities are at their saturation capacity for the provision of clinical care, especially regarding ICU beds and mechanical ventilators^8–20^. Data-driven solutions are needed to support decision-making^11^.

COVID-19 has shown to rapidly worsen a few days after infection^12,13^. The median time from disease onset to ICU admission is 9–12 days^14,15^. About 26–32% of the hospitalized patients are eventually admitted to ICU, and mortality in this group ranges from 39 to 72%, depending on the local characteristics of patients^14,15^. The median length of ICU stay and use of mechanical ventilation is approximately 9 days (95% CI 6.5–11.2) and 8.4 days (95% CI 1.6–13.7), respectively^16^.

Previous studies have used blood tests^17^, CT images^18,19^, sociodemographic and comorbidities history^20^ to develop COVID-19 diagnostic and prognostic models, including machine learning techniques^21–23^. Biomarkers from blood tests have emerged as important variables for poor prognostic factors^24^, which are a promising tool in poorer regions, due to its low cost and inclusion in standard protocols for clinical care. However, the majority of studies^25^ rely on algorithms trained on a single prognostic outcome, which in theory require the training of specific algorithms for each distinct negative outcome.

This study proposes to develop multipurpose machine learning algorithms to analyze if it is possible to predict overall poor prognosis for COVID-19 patients. We aim to test if the algorithms can generalize risk patterns for severe conditions, so they can be used as tools to assist in the prognosis of distinct negative outcomes for COVID-19 patients.

## Results

### Descriptive statistics

Table 1 shows the descriptive statistics for the demographic characteristics of the patients. The sample of the study (1040 patients with COVID-19) was mostly comprised by men (53.3%), with an average age of 51.7 years, and the majority of patients (63.8%) were white. The full descriptive statistics for all variables are presented in Supplementary Table 1.

**Table 1:** Descriptive statistics of the demographics characteristics of the sample, BP Hospital—A Beneficência Portuguesa de São Paulo, Brazil, 2020.

Variable	ICU	MV	Death	Total
	Mean (SD)	Mean (SD)	Mean (SD)	Mean (SD)
Age (years)	63.2 (17.1)	65.4 (16.2)	73.7 (14.4)	51.7 (18.9)
Weight (kg)	79.80 (17.8)	78.9 (16.4)	74.5 (12.0)	80.9 (18.7)
BMI	28.2 (5.4)	27.8 (4.4)	27.1 (4.1)	28.8 (5.9)
Height (cm)	146.1 (56.5)	147.9 (55.9)	152.4 (47.1)	154.9 (44.0)
**Gender**				
Female (%)	42.0	34.9	42.4	46.7
**Race (%)**				
Asian	1.4	0.9	1.1	1.2
White	71.2	72.6	81.5	63.8
Indigenous	0.4	0.9	1.1	0.2
Black	3.6	1.9	1.1	3.2
Mixed	16	20.8	13	14.1
N/A	7.5	2.8	2.2	17.5

### Algorithms performance

We analyzed the predictive performance of the algorithms for three negative prognostic outcomes: ICU admission (n = 263, 25.5%), mechanical ventilation (MV) intubation (n = 106, 10.2%) and death (n = 92, 9.4%).

First, we tested the predictive performance of the machine learning algorithms for a specific individual outcome (e.g. death) to get a baseline for comparison. Then, we used observations from patients who had the other two outcomes (in this specific example, mechanical ventilation and ICU admission) to train an aggregated model. In the aggregated model, we tested the performance when predicting the severe outcome not included in training (e.g. death). Finally, we compared the performance of the two strategies (e.g. individual against aggregated models) using the 95% confidence interval of the area under the receiver operating characteristic curve (AUROC).

Table 2 shows the results of the models trained with the aggregated outcomes and the models with a single outcome. Every model, even the ones trained with different outcomes, presented high predictive performance, always with an AUROC over 0.91 in the test set. The individual models presented better AUC compared to the aggregated models when predicting ICU, MV or death with AUROC over 0.959, 0.945 and 0.972 respectively.

**Table 2:** Predictive performance comparison in the test set for aggregated and individual models, BP Hospital—A Beneficência Portuguesa de São Paulo, Brazil, 2020.

Combination	Best algorithm	AUC [95% C.I.]	Sensitivity	Specificity	PPV	NPV	F1
**ICU + MV**							
Predict ICU	*Random forest*	0.959 [0.94; 098]	0.906	0.868	0.720	0.961	0.802
Predict MV		0.912 [0.87; 0.95]	0.935	0.723	0.271	0.990	0.420
Predict death		0.925 [0.89; 0.96]	0.969	0.730	0.290	0.995	0.446
**Only death**							
Predict death	*Extra trees*	0.972 [0.95; 1.00]	0.964	0.863	0.409	0.996	0.574
**ICU + death**							
Predict ICU	*XGBoost*	0.965 [0.95; 0.98]	0.847	0.930	0.818	0.942	0.832
Predict MV		0.925 [0.89;0.96]	0.946	0.808	0.398	0.991	0.560
Predict Death		0.922 [0.89; 0.95]	1.000	0.787	0.307	1.000	0.470
**Only MV**							
Predict MV	*Extra trees*	0.945 [0.91;0.98]	0.906	0.819	0.362	0.987	0.518
**MV + death**							
Predict ICU	*Random forest*	0.921 [0.89; 0.95]	0.765	0.901	0.729	0.917	0.747
Predict MV		0.940 [0.91; 0.97]	0.933	0.799	0.329	0.991	0.487
Predict death		0.943 [0.91; 0.98]	0.963	0.794	0.306	0.996	0.464
**Only ICU**							
Predict ICU	*Random forest*	0.959 [0.94; 0.98]	0.906	0.868	0.720	0.961	0.802

Despite the individual models being overall better, the difference between the aggregated and individual models were all within the 95% confidence intervals. Supplementary Fig. 1 shows the AUROC for each model. The sensitivity and specificity of the machine learning algorithms were also very high, in most cases over 0.8, with an average sensitivity of 0.92 and specificity of 0.82.

The positive predictive values (PPV) for the aggregated models were higher than the individual models when predicting mechanical ventilation and ICU, reaching 0.398 and 0.729 respectively, while for death there was a decrease to 0.290. This means that two out of three of the aggregated models had higher PPV when predicting which patients would develop severe illness and require hospital resources than the individual models. In Supplementary Table 2 we present the final hyperparameters for each model.

### Interpretability

**Figure 1:**
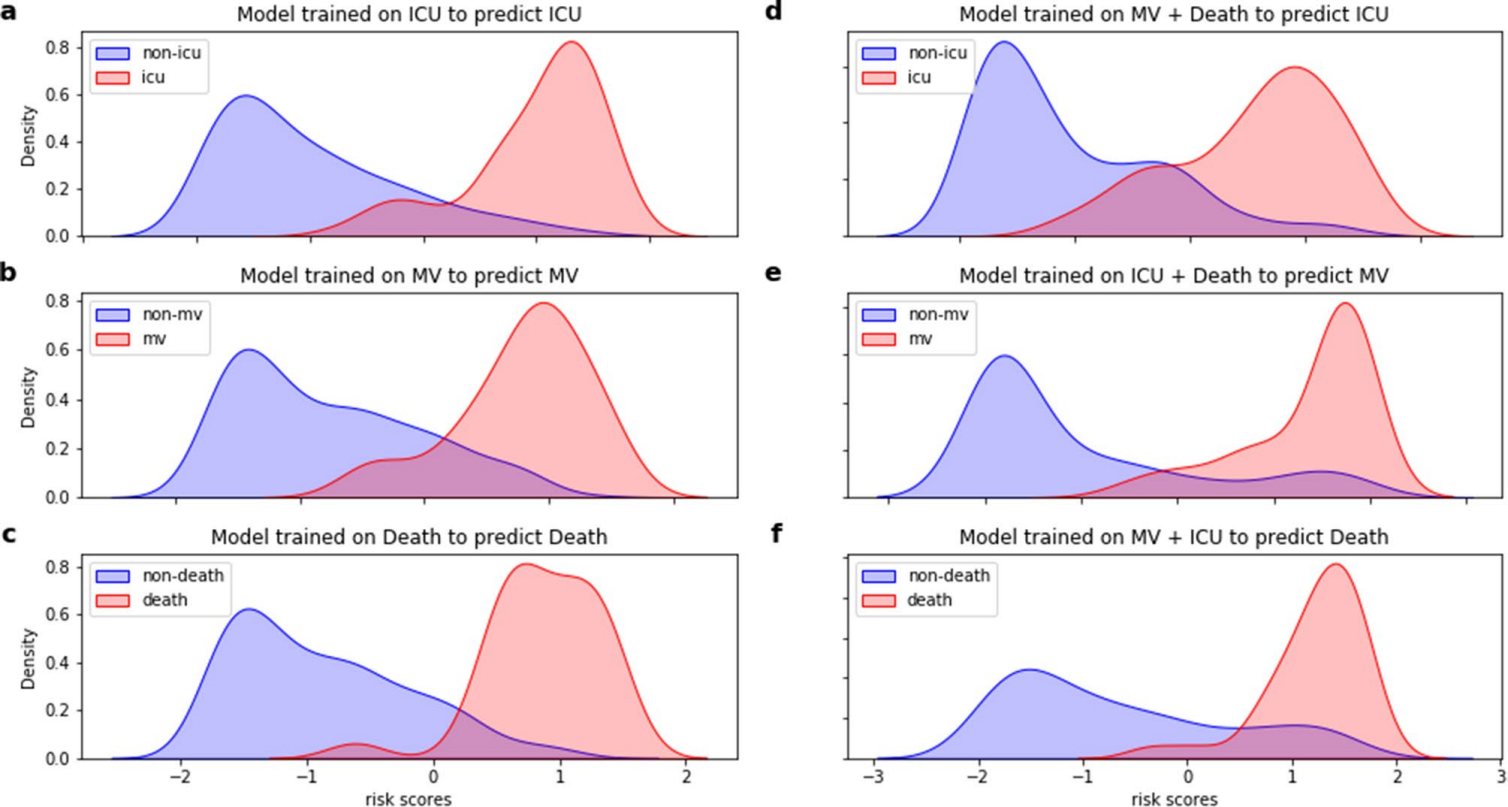
Density plots for the three severe COVID-19 outcomes, BP Hospital—A Beneficência Portuguesa de São Paulo, Brazil, 2020. (**a**–**c**) Density plots for the single outcome models. (**d**–**f**) Density plots for the aggregated models predicting unspecific outcome.

Figure 1 presents the prediction density for each individual outcome according to the different training strategies. The results point to a low overlap between negative and positive cases, indicating a good discriminative ability of the algorithms irrespective of the training strategy.

**Figure 2:**
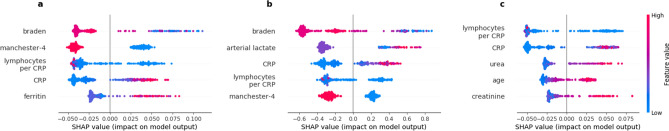
Top five feature contributions to predict severe outcome in the aggregated models, BP Hospital—A Beneficência Portuguesa de São Paulo, Brazil, 2020. (**a**) Combined outcomes (MV + ICU) to predict death (**b**) Combined outcomes (Death + ICU) to predict MV. (**c**) Combined outcomes (Death + MV) to predict ICU.

Figure 2 presents the top five variables that most contributed to predict a severe outcome in the aggregated models, according to the Shapley values. The variables are ranked according to the contribution for each specific algorithm. The Braden score played an important role in the aggregated outcome algorithms, ranking as the most important predictor in two of the three models. Also, the C-reactive protein and ratio of lymphocytes per C-reactive protein were found to be good predictors, appearing in the top five in all three models. Urea, age, creatinine, and arterial lactate were important for only one of the aggregated models.

## Discussion

Previous studies have used machine learning to develop early COVID-19 prognostic models for a specific severe outcome with overall good performance^21,23^, frequently reaching over 0.90 AUROC^26^. We used a different approach, by combining severe outcomes to train algorithms to predict another outcome, in order to test its potential for predicting multiple untrained outcomes.

We found that machine learning algorithms were able to predict negative prognostic outcomes with high overall performance for COVID-19, even when the specific outcome was not included in the training of the algorithms. All models presented an AUROC higher than 0.91 (average of 0.92) in the test set, with high sensitivity and specificity (average of 0.92 and 0.82, respectively). The results highlight the possibility that high-performance machine learning algorithms are able to predict unspecific negative COVID-19 outcomes using routinely-collected data.

The development of multipurpose prognostic algorithms, i.e. algorithms that identify nonspecific outcomes and overall future clinical deterioration, can be used in a large number of situations, especially in the case of complex and unknown diseases that lead to the development of several different negative outcomes. Instead of having to develop a different algorithm for each of the specific outcomes, multipurpose models can provide more comprehensive and clinically relevant information about the risks of future health problems of patients. The algorithms can be embedded in an app for smartphones or in electronic medical records to be used with routinely-collected data to perform simple predictions for each incoming patient, thus supporting screening procedures and decision-making. In the case of developing countries, while the issue of current availability of electronic medical records in poorer areas is still a challenge, in Brazil there have been promising recent advances regarding the use of electronic medical records^27^.

Brazil is currently the third country in the world in total number of cases and second in deaths from COVID-19^28^. There is a growing demand in Brazil, and in many other developing countries, for decision support in the allocation of scarce hospital resources, especially in relation to the availability of ICU beds and mechanical ventilators^29,30^. From a clinical standard, knowledge about immediate risks of negative prognosis can also contribute to the early start of preventive measures and new interventions, and thereby increase patient survival^5,6^.

For every outcome, variable importance analysis identified that age, C-reactive protein (CRP), creatinine, urea and the Braden Scale were usually among the most important. While the age of the patient is widely found to be an important predictor for most negative health outcomes, CRP has been increasingly included among the main inflammatory biomarkers for the prognosis of cardiovascular^31^ and respiratory diseases^32^. High levels of CRP have been also previously associated with individual severity of SARS-CoV-2^33,34^. Interestingly, previous studies have also identified that chronic kidney disease is associated with developing severe conditions in COVID-19 patients^35–37^, where it has been observed that patients with higher levels of creatinine and urea are more at risk^38^. The Braden Scale is often used as a predictor for pressure ulcers, a common clinical classification scale for predicting pneumonia^39^ during clinical reception, and in this study, it was an important predictor for negative prognosis in COVID-19 patients. The scale has a score between 1 (worst score) and 4 (best score) where the factors included are sensory perception, skin moisture, activity, mobility, nutritional status and friction^40^. The percentage of lymphocytes in the blood has been described as a strong predictor of prognosis for the severity of the new coronavirus. A randomized study by Tan et al.^41^ suggested that, in most confirmed cases, the percentage of lymphocytes was reduced to 5% in 2 weeks after the onset of COVID-19, in line with other studies findings^42^.

The study has a few limitations that need to be mentioned. First, some of the outcomes overlap, which may have helped the performance of the aggregated models, even though in the majority of cases the outcomes were independent. In the case of ICU admission, 55% of the patients did not die or used MV, while in the case of MV and death, 63% and 70% of their respective aggregated model was trained on other outcomes. Ideally, the outcomes would never overlap, but this is clinically unfeasible given the interlaced nature of negative prognostic outcomes. Another limitation is that we analyzed data from an urban COVID-19 hotspot in Brazil, in a period where clinical protocols for the disease were still being established, so this could affect the incidence of prognostic outcomes and may not directly generalize to other periods.

In conclusion, we found that machine learning algorithms can predict severe outcomes in COVID-19 patients with high performance, including previously unobserved outcomes, using only routinely-collected laboratory, clinical and demographic data. The use of multipurpose algorithms for the prediction of overall negative prognosis is a promising new area that can support doctors with clinical and administrative decisions, especially regarding priorities for hospital admission and monitoring.

## Methods

### Data source

We followed a cohort of 3280 patients with a RT-PCR diagnostic exam for COVID-19 from a large hospital chain in the city of São Paulo (BP-A Beneficência Portuguesa de São Paulo) between March 1, 2020, and 28 June, 2020. Of these, 1040 (31.7%) patients were positive for COVID-19 and were included in the analysis. The study was approved by the Institutional Review Board (IRB) of BP—A Beneficência Portuguesa de São Paulo (CAAE:31177220.4.3001.5421), including a waiver of informed consent. The study followed the guidelines of the transparent reporting of a multivariable prediction model for individual prognosis or diagnosis (TRIPOD)^43^.

**Figure 3:**
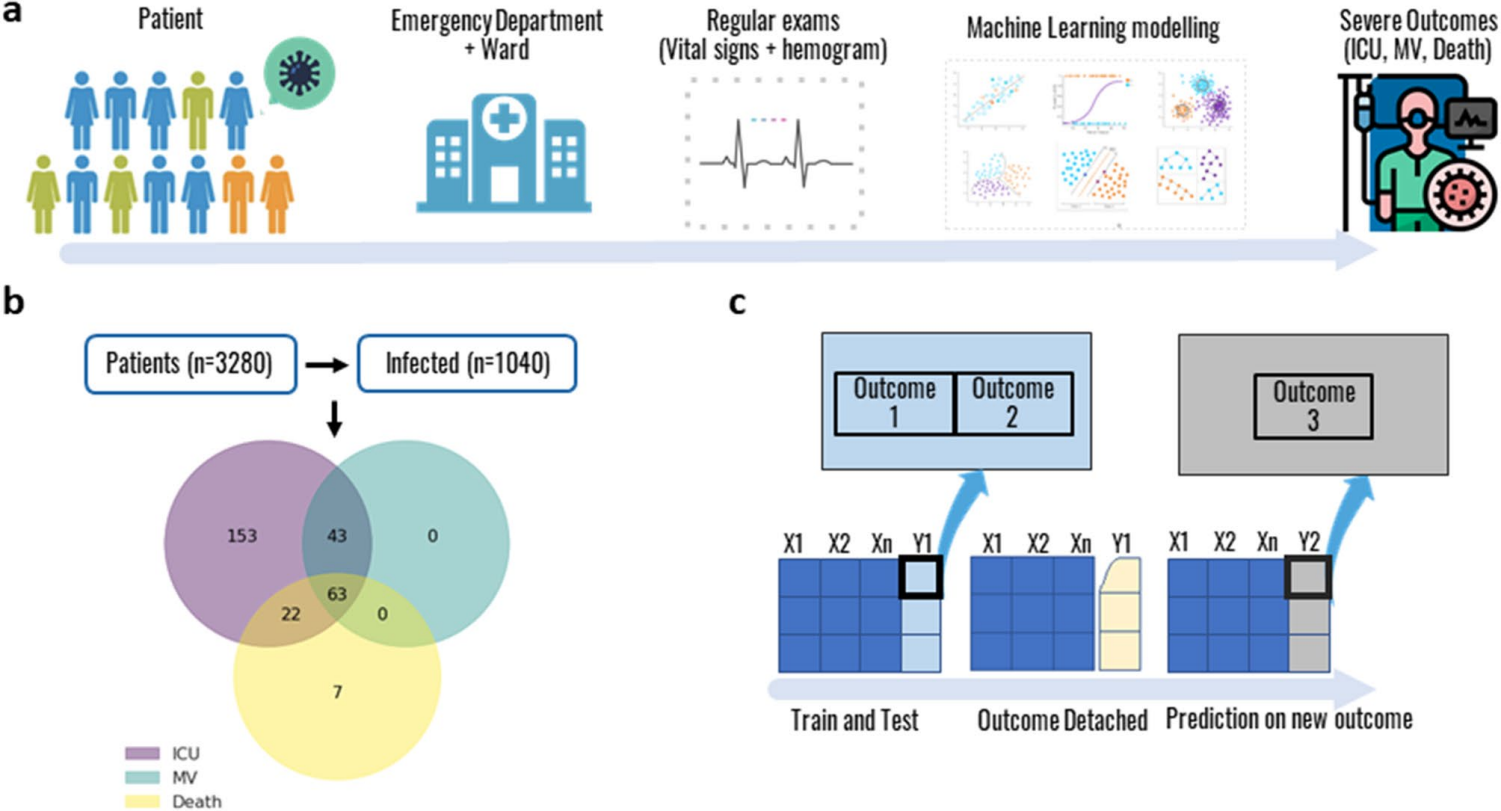
Overview of the study process. (**a**) From hospital admission to the final outcome. (**b**) Population inclusion criteria and outcomes intersection. (**c**) The algorithm was trained and tested using a combination of two outcomes. The same algorithm was then used to predict the remaining outcome.

Individual patient data was collected from electronic medical records. We included as predictors only variables collected in early hospital admission, i.e. within 24 h before and 24 h after the RT-PCR exam. From a total of 82 routinely-collected variables from the hospital, 57 variables were selected for the development of the predictive models, after removing variables with 90% or higher missing values, highly-correlated variables (above 0.9) and identifying variables such as patient number and hospital identification variables. The flowchart for feature selection is described in Supplementary Fig. 2 and the complete variable list, including demographic data, laboratory tests and vital signs is described in Supplementary Table 1. Figure 3 illustrates the overall process.

### Machine learning techniques

Five of the most popular machine learning models for structured data (artificial neural networks^44^, extra trees^45^, random forests^46^, catboost^47^, and extreme gradient boosting^48^) were trained with 70% of the data, and tested in the other 30%, simulating new unknown data. All the results reported in this study are from the test set. K-fold cross-validation with 10 folds was used to adjust the hyperparameters with Bayesian optimization (HyperOpt). Due to the unbalanced nature of the outcomes, random undersampling was performed in the training set, by randomly selecting examples from the majority class for exclusion. This technique was implemented using the RandomUnderSampler imbalanced-learn class^49^.

Variables with more than two categories were represented by a set of dummy variables, with one variable for each category. Continuous variables were standardized using the z-score. Variables with a correlation greater than 0.90 (mean arterial pressure, total bilirubin, and creatine kinase) were discarded, and missing values were imputed by the median. To assess the performance of the models, measures such as accuracy, sensitivity (also known as recall), specificity, positive predictive value (PPV) (also known as precision), negative predictive value (NPV), and F1 score were analyzed. The value of the AUROC was used to select the best model. To understand the individual contribution of each variable to the predictive models, we calculated their respective Shapley values. All the analyzes were performed using the Python programming language with the scikit-learn library.

## Supplementary Information

Supplementary Information.

## Data Availability

The data comes from electronic medical records from BP—A Beneficência Portuguesa de São Paulo Hospital in Brazil and it is not publicly available as it contains sensitive information of patients.

## Code availability

All the code written to process and analyze the data can be made available upon request to the corresponding author.
