# Supplementary material for: A multipurpose machine learning approach to predict COVID-19 negative prognosis in São Paulo, Brazil
Source: Sci Rep. 2021 Feb 8;11:3343. doi: 10.1038/s41598-021-82885-y (PMC7870665; doi:10.1038/s41598-021-82885-y)
Supplement: Supplementary file 1 — Supplementary Information. [file 41598_2021_82885_MOESM1_ESM.docx]

**A multipurpose machine learning approach to predict COVID-19 negative prognosis in São Paulo, Brazil**

***Fernando Timoteo Fernandes^1,2^**

**Tiago Almeida de Oliveira^1,3^**

**Cristiane Esteves Teixeira^1,4^**

**Andre Filipe de Moraes Batista^1^**

**Gabriel Dalla Costa^5^**

**Alexandre Dias Porto Chiavegatto Filho^1^**

**^1^ School of Public Health, University of São Paulo, SP, Brazil**

**^2^ Fundacentro, São Paulo, SP, Brazil**

**^3^ Statistics Department, Paraíba State University, Paraíba, PB, Brazil**

**^4^ Brazilian National Cancer Institute, Bioinformatics and Computational Biology Lab, Rio de Janeiro, RJ, Brazil**

**^5^ BP - A Beneficência Portuguesa de São Paulo, SP, Brazil**

**Correspondence to:** [**fernando.fernandes@fundacentro.gov.br**](mailto:fernando.fernandes@fundacentro.gov.br)

**Supplementary Table 1 -** Descriptive statistics of model variables.

| **Variable (unit)** | **ICU** | **MV** | **Death** | **Total** |
| --- | --- | --- | --- | --- |
| **Demographics** | **Mean (SD)** | **Mean (SD)** | **Mean (SD)** | **Mean (SD)** |
| Age (years) | 63.2 (17.1) | 65.4(16.2) | 73.7 (14.4) | 51.7 (18.9) |
| Weight (kg) | 79.80 (17.8) | 78.9 (16.4) | 74.5 (12.0) | 80.9 (18.7) |
| BMI | 28.2 (5.4) | 27.8(4.4) | 27.1 (4.1) | 28.8 (5.9) |
| Height (cm) | 146.1 (56.5) | 147.9 (55.9) | 152.4 (47.1) | 154.9 (44.0) |
| Gender | **n (%)** | **n (%)** | **n (%)** | **n (%)** |
| Male (%) | 163 (58.0) | 69 (65.1) | 53 (57.6) | 554 (53.3) |
| Race |  |  |  |  |
| 1. Asian | 4 (1.4) | 1 (0.9) | 1 (1.1) | 13 (1.2) |
| 1. White | 200 (71.2) | 77 (72.6) | 75 (81.5) | 663 (63.8) |
| 1. Indian | 1 (0.4) | 1 (0.9) | 1 (1.1) | 2 (0.2) |
| 1. Black | 10 (3.6) | 2 (1.9) | 1 (1.1) | 34 (3.2) |
| 1. Brown | 45 (16.0) | 22 (20.8) | 12 (13.0) | 146 (14.1) |
| N/A | 21 (7.5) | 3 (2.8) | 2 (2.2) | 182 (17.5) |
| **Vital Signs** | **Mean (SD)** | **Mean (SD)** | **Mean (SD)** | **Mean (SD)** |
| Heart Rate (Beats per minute) | 91.1(16.6) | 91.4 (17.1) | 91.8 (18.1) | 90.0 (14.3) |
| Respiratory Rate (Breaths per minute) | 19.8(3.9) | 20.4(4.8) | 20.9 (5.0) | 18.5 (2.9) |
| Temperature (Celsius Degree) | 36.8 (0.8) | 36.7(0.8) | 36.8 (0.8) | 36.7 (0.7) |
| Systolic blood pressure (mmHg) | 126.8 (23.4) | 126.0(25.7) | 123.5 (26.5) | 129.4 (19.0) |
| Diastolic blood pressure (mmHg) | 74.7 (13.9) | 72.9 (14.3) | 70.6 (15.8) | 79.5 (11.8) |
| Oxygen Saturation (mmHg) | 92.5 (6.9) | 91.7(9.1) | 92.5 (6.8) | 95.3 (4.8) |
| **Blood Measures (Hemogram)** |  |  |  |  |
| Hemoglobin (g/dL) | 12.6 (2.2) | 12.1 (2.3) | 89.8 (11.5) | 13.5 (1.8) |
| Platelet count (/mm3) | 203170.8 (88497.2) | 195971.6 (89697.8) | 181521.739 (83047.6) | 207422.1 (77468.1) |
| Red cells count (/mm3) | 4.3 (0.7) | 4.1 (0.8) | 4.0 (0.9) | 4.6 (0.6) |
| Mean Corpuscular volume (fL) | 87.6 (6.3) | 88.9 (6.8) | 89.3 (7.0) | 86.3 (5.2) |
| Mean Corpuscular Hemoglobin (pg) | 29.1 (2.2) | 29.1 (2.2) | 29.2 (2.3) | 29.0 (1.9) |
| Mean Corpuscular Hemoglobin concentration (g/dL) | 33.2 (1.1) | 32.8 (1.1) | 32.7 (1.3) | 33.6 (1.1) |
| Red cell distribution width (%) | 14.1 (1.9) | 14.6 (2.2) | 15.3 (2.6) | 13.4 (1.5) |
| Leukocytes Count (/mm3) | 8228.8 (4898.6) | 9677.6 (6269.0) | 8767.935 (5122.4) | 6594.5 (3475.5) |
| Neutrophil Count (/mm3) | 7280.5 (4795.7) | 7699.7 (5054.4) | 6394.6 (4218.3) | 4486.3 (3093.2) |
| Basophil Count (/mm3) | 17.6 (16.2) | 17.3 (16.5) | 16.3 (16.0) | 20.1 (17.8) |
| Eosinophil Count (/mm3) | 47.8 (109.3) | 46.6 (118.1) | 36.3 (94.3) | 74.7 (233.1) |
| Monocyte Count (/mm3) | 585.8 (396.2) | 620.9 (501.6) | 544.7 (377.2) | 588.1 (309.1) |
| Lymphocyte count (/mm3) | 1212.6 (2214.3) | 1385.9 (3555.5) | 986.2 (670.8) | 1431.1 (1295.7) |
| C Reactive Protein (mg/dL) | 10.3 (9.0) | 13.8 (10.8) | 14.4 (10.9) | 4.7 (6.7) |
| Creatinine (mg/dL) | 1.5(1.4) | 1.8 (1.7) | 2.0 (1.7) | 1.1 (0.9) |
| Blood Sodium (mEq/L) | 137.2 (4.6) | 137.7 (5.7) | 137.5 (6.5) | 137.9 (3.7) |
| Potassium  (mEq/L) | 4.0 (0.6) | 4.2 (0.6) | 4.2 (0.6) | 4.0 (0.4) |
| Lactate dehydrogenase level (U/L) | 685.9 (309.9) | 814.7 (349.6) | 795.4 (372.2) | 540.3 (244.6) |
| D Dimer (ng/dL) | 2498.4 (7378.8) | 3607.8 (11016.0) | 4723.793 (12336.0) | 1363.4 (4615.1) |
| Alanina aminotransferase (U/L) | 52.2 (99.9) | 53.8 (85.1) | 54.7 (37.1) | 43.5 (71.5) |
| Aspartate aminotransferase (U/L) | 56.8 (72.0) | 65.5 (81.3) | 36.9 (34.4) | 43.9 (52.5) |
| Direct Bilirubin (mg/dL) | 0.3 (0.3) | 0.3 (0.3) | 0.3 (0.3) | 0.2 (0.2) |
| Indirect Bilirubin (mg/dL) | 0.1(0.1) | 0.1 (0.1) | 0.1 (0.1) | 0.1 (0.1) |
| Blood Urea (mg /dL) | 52.8(39.2) | 66.0 (45.7) | 76.0 (48.1) | 38.0 (26.6) |
| Troponin (mcg/L) | 4.3(36.9) | 4.5 (29.3) | 0.7 (2.6) | 2.0 (24.1) |
| Venous Lactate (mmol/L) | 15.0(6.0) | 15.6 (4.4) | 15.7 (6.3) | 14.9 (6.0) |
| Arterial Lactate (mmol/L) | 14.6(8.6) | 16.3 (11.4) | 17.2 (11.1) | 14.1 (8.0) |
| Creatine Phosphokinase (U/L) | 297.8(797.5) | 468.3 (1187.9) | 277.0 (690.3) | 197.7 (540.0) |
| Activated Partial Thromboplastin Time (seconds) | 1.1 (0.4) | 1.1 (0.4) | 1.1 (0.4) | 1.1 (0.4) |
|  |  |  |  |  |
| **Arterial Blood Gas** |  |  |  |  |
| pH | 7.4 (0.0) | 7.3 (0.0) | 7.4 (0.08) | 7.4 (0.1) |
| Partial pressure of oxygen - PaO2 (mmHg) | 109.1(49.3) | 115.9 (51.4) | 114.6 (47.9) | 107.6 (45.1) |
| Partial pressure of carbon dioxide - PaCO2 (mmHg) | 35. 8 (8.5) | 38.2 (10.6) | 36.5 (10.0) | 35.0 (7.2) |
| Bicarbonate - HCO3 (mEq/L) | 22.6 (3.7) | 22.6 (4.0) | 22.0 (4.4) | 22.8 (3.1) |
| Base Excess (mEq/L) | -0.9 (3.7) | -1.5 (3.8) | -1.8 (4.3) | -0.4 (2.9) |
| Oxygen saturation - sO2 (%) | 93.6(11.5) | 93.7 (12.7) | 94.1 (12.2) | 94.1 (10.9) |
| Magnesium (mmol/L) | 2.1 (0.3) | 2.2 (0.3) | 2.1 (0.3) | 2.1 (0.3) |
| Calcium ionised (mmol/L) | 1.1(0.0) | 1.1 (0.0) | 1.1 (0.09) | 1.1 (0.1) |
| Glucose (mg/dL) | 155.0 (82.2) | 181.6 (91.6) | 180.0 (91.7) | 140.5 (69.3) |
| **Scales and Derived measures** |  |  |  |  |
| Braden  <9  9-12  13-14  15-18  19-23  N/A | **n (%)**  9 (3.4)  37 (14.2)  39 (14.9)  62 (23.8)  112 (42.9)  2 (0.8) | **n (%)**  7 (6.6)  26 (24.5)  11 (10.4)  21 (19.8)  40 (37.7)  1 (0.9) | **n (%)**  7 (7.6)  22 (23.9)  14 (15.2)  20 (21.7)  27 (29.3)  2 (2.2) | **n (%)**  11 (1.2)  45 (4.3)  41 (3.9)  95 (9.1)  352 (33.8)  496 (47.7) |
| International Normalized Ratio (INR) | 1.2 (0.3) | 1.2 (0.3) | 1.2 (0.3) | 1.1 (0.2) |
| Neutrophil per lymphocyte (ratio)  Lymphocyte per C reactive protein (ratio) | 7.9 (8.0)  606.8 (2215.4) | 10.4 (10.1)  292.1 (830.7) | 10.6 (10.5)  184.5 (407.2) | 4.4 (5.2)  4787.1 (14367.2) |
| Manchester  1  2  3  4  5  N/A | **n (%)**  3 (1.1)  61 (21.7)  89 (31.7)  47 (16.7)  2 (0.7)  79 (28.1) | **n (%)**  2 (1.9)  23 (21.7)  28 (26.4)  16 (15.1)  0.0 (0.0)  37 (34.9) | **n (%)**  2 (2.2)  27 (29.3)  24 (26.1)  12 (13.0)  0.0 (0.0)  27 (29.3) | **n (%)**  4 (0.4)  89 (8.5)  182 (17.5)  572 (55.0)  7 (0.7)  186 (17.8) |

**Supplementary Table 2 –** Hyperparameters of the best machine learning algorithms for each outcome and training combination.

| **Combination** | **Best algorithm** | **Hyperparameters** |
| --- | --- | --- |
| **ICU + MV**  *predict ICU* | *Random Forest* | n_estimators = 935, max_depth=406  max_features='log2', min_samples_leaf=2  min_samples_split=36 |
| *predict MV* |  |  |
| *predict Death* |  |  |
| **Only Death** | *Extra Trees* | max_depth=100, min_samples_leaf=4  n_estimators=135 |
| **ICU + Death** | *XGBoost* | base_score=0.5, colsample_bylevel=1  colsample_bynode=1, colsample_bytree=0.55  gamma=0.65, importance_type='gain'  learning_rate=0.075  subsample=0.55  max_delta_step=0, max_depth=6  min_child_weight=3.0  missing=nan  n_estimators=75, n_jobs=-2  num_parallel_tree=1, reg_alpha=1.25  reg_lambda=1  scale_pos_weight=1 |
| *predict ICU* |  |  |
| *predict MV* |  |  |
| *predict Death* |  |  |
| **Only MV** | *Extra Trees* | max_depth=20, min_samples_leaf=4  n_estimators=135 |
| **MV + Death** | *Random Forest* | n_estimators = 935, max_depth=406, min_samples_split=36, max_features='log2', min_samples_leaf=2 |
| *predict ICU* |  |  |
| *predict MV* |  |  |
| *predict Death* |  |  |
| **Only ICU** | *Random Forest* | max_depth=406, max_features='log2', n_estimators=935 min_samples_leaf=2, min_samples_split=36 |


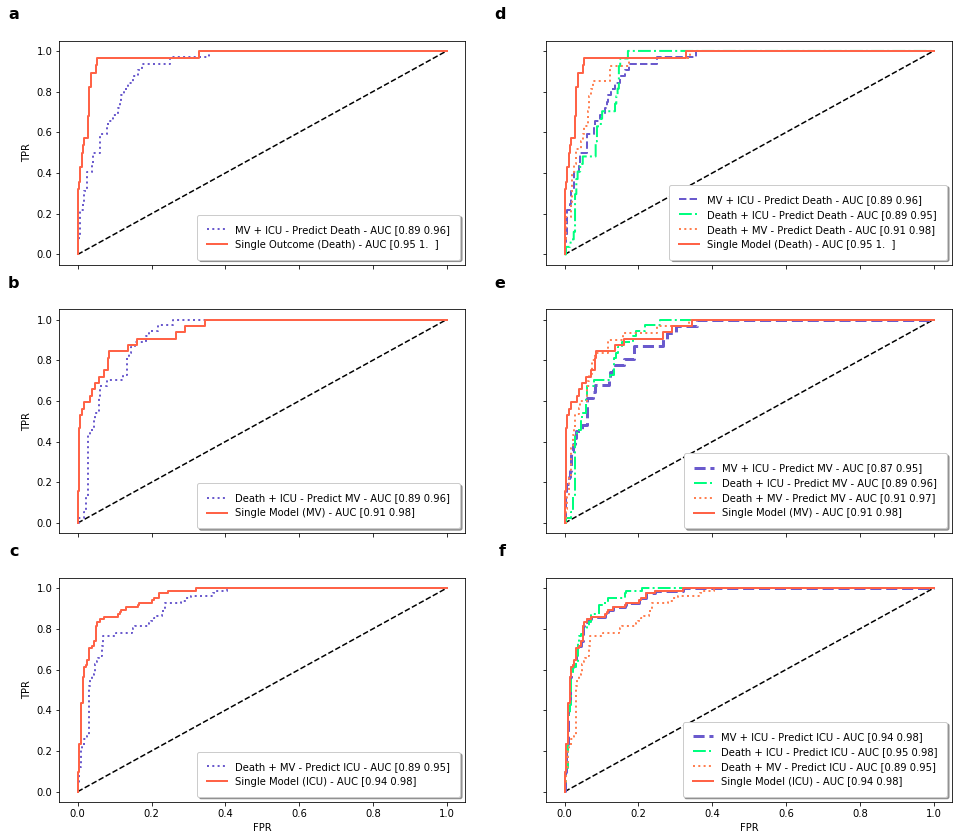


**Supplementary Figure 1.** Area under the ROC curves comparison on all possible combinations.

**
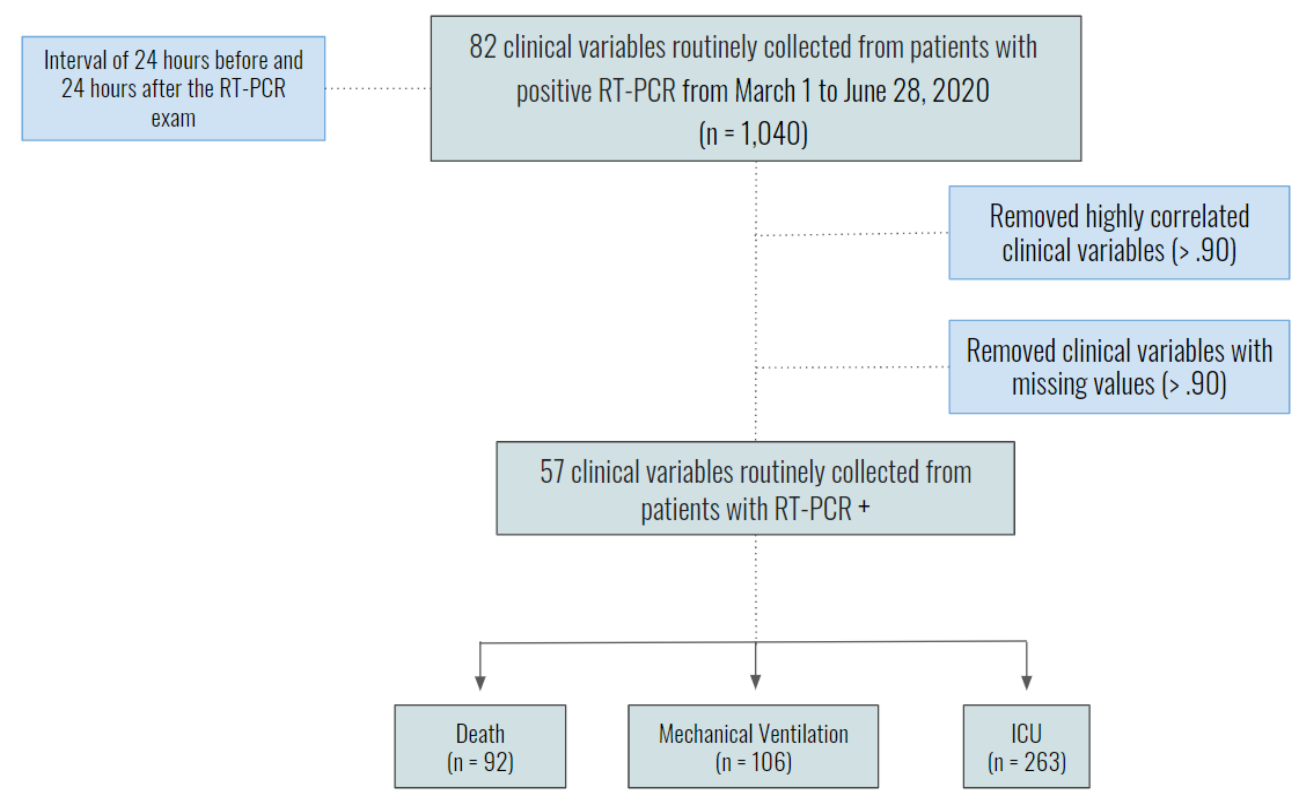
**

**Supplementary Figure 2.** Feature selection and outcome distribution. From initial routinely-collected variables to final variables used in the developed models.
